# Augmented Reality in Minimally Invasive Spinal Interventions: Current Use and Future Directions

**DOI:** 10.7759/cureus.80119

**Published:** 2025-03-05

**Authors:** Anika Pruthi, Theres Alexander, Aizhan Mengaliyeva, Nikhil Kota, Manisha Koneru

**Affiliations:** 1 Neurosciences, Cooper Medical School of Rowan University, Camden, USA; 2 Neurointerventional Surgery, Cooper University Health Care, Camden, USA

**Keywords:** augmented reality (ar), learning curve, minimally invasive and robotic spine surgery, minimally-invasive spine, simulation in medical education

## Abstract

Minimally invasive spinal interventions have a steep learning curve from a technical perspective, as they are associated with performing precise maneuvers where the surgeon may or may not have direct or full visualization of the patient’s anatomy. Augmented reality (AR), where models of the patient’s anatomy can be overlaid within the surgical field, has offered promise to improve the operative experience. We present a qualitative review of recent advances in applications of AR technology in minimally invasive spinal procedures in both clinical and educational settings. We explore current evidence of experiences with this technology and highlight key areas for future development. Through this review, we aim to provide a deeper understanding of the current state of AR in transforming both the clinical and educational realms of minimally invasive spinal surgery.

## Introduction and background

Technological advances in spinal surgery have mitigated many postoperative concerns associated with traditional open surgical approaches. Using small incisions to access the spine, minimally invasive surgical spine (MISS) techniques are able to reduce soft tissue injuries, decrease postoperative pain, and limit blood loss [1]. When considering common MISS procedures such as lumbar puncture, spinal biopsy, vertebroplasty, kyphoplasty, and radiofrequency ablation, patients value having expanded therapeutic options that offer faster recovery and improved cosmesis [2,3].

While it is beneficial that these techniques are less invasive, they can present significant technical challenges [4]. MISS relies on precision using specialized instruments and advanced tools involving robotics and lasers. With this steep technical learning curve, limited visualization, and complex anatomical structures, the mastery of these techniques can be difficult for even the most experienced clinicians. Augmented reality (AR) technology can help surgeons with the precision and accuracy required in these MISS interventions by overlaying virtual elements in the real-world environment. With its ability to provide enhanced visualization and spatial guidance, AR can be applied beyond procedural settings as an educational tool as well.

This review will describe the currently available AR products for minimally invasive spinal interventions, applications of AR in these settings, and areas for future directions.

## Review

Overview of AR systems

AR is a software system that projects 3D holographic images of anatomical structures directly onto the surgical field. Surgeons are able to visualize the images of structures, such as pedicles and discs, through specialized headsets or glasses without having to take their eyes off of the patient. In addition to proprietary headsets, commercial headsets have been used to display surgical AR visualizations. The wireless Microsoft HoloLens^®^ (Microsoft Corporation, Redmond, WA, USA) display headset is a mixed-reality system that is voice controlled, incorporates hand tracking, and allows for adjustments to interpupillary distance [5]. The Apple Vision Pro^™^ (Apple Inc., Cupertino, CA, USA) and Meta Quest^®^ (Meta Platforms, Inc., Menlo Park, CA, USA) display headsets are other mixed reality systems with similar features. Yet, the limited battery life and bulkier frames can pose challenges in the preoperative planning workflow and intraoperative use [6].

Several AR systems are currently being used or explored for MISS, as summarized in Table 1. Various AR systems are commonly applied for pedicle screw placement [7]. However, these systems differ in design, cost, and functionality. The first AR system approved for preoperative planning for MISS was OpenSight^®^ (Novarad, Provo, UT, USA). XVision^®^ (Novarad) has a surgical tracking system and display that projects all the graphical details onto the surgeon’s retina but requires intraoperative imaging CT scans for the overlay [6]. The use of registration markers in the system to align the virtual anatomy elements with the real-time environment can also become a limitation when markers may not work as effectively in rigid or irregular anatomical locations. ImmersiveAR™ (ImmersiveTouch Inc., Chicago, IL, USA) technology has been incorporated into intraoperative and educational settings [5]. VisAR^®^ (Novarad), an AR visor, is precise and uses low-cost hardware but also relies on preoperative scans being available. However, pending FDA approval, SurgicalAR^®^ (Medivis, New York, NY, USA) incorporates artificial intelligence-driven landmark registration to mitigate issues with inaccurate models with features to aid preoperative and intraoperative needs.

**Table 1:** Currently available AR systems for minimally invasive spine surgery AR, augmented reality

Name	Company	FDA approval status	Year of approval	Procedural applications
OpenSight^®^	Novarad (Provo, UT, USA)	501(k) clearance	2018	Preoperative planning, spinal injection, percutaneous vertebroplasty, kyphoplasty, pedicle screw navigation, and spinal rod implant
XVision^®^	Augmedics Inc. (Arlington Heights, IL, USA)	501(k) clearance	2021	Pedicle screw placement and osteotomy
VisAR^®^	Novarad (Provo, UT, USA)	501(k) clearance	2022	Intraoperative pedicle screw implementation and neurosurgical navigation
ImmersiveAR^™^	ImmersiveTouch Inc. (Chicago, IL, USA)	501(k) clearance	2024	Pedicle screw implementation
SurgicalAR^®^	Medivis (New York, NY, USA)	Pending	-	Pedicle screw and interbody placement

Clinical applications of AR in MISS

The integration of AR in MISS shows great promise in optimizing clinical treatment outcomes and procedural accuracy. Like virtual reality, AR projects overlays to differentiate anatomical landmarks and expand the surgeon’s field of view [8]. These models can help assess potential risks and provide real-time augmented feedback to influence surgical recommendations, particularly for challenging cases. For example, Bardeesi et al. (2024) report using Smartbrush (Brainlab AG, Munchen, Germany) to create a preoperative 3D model for a patient with spondylolisthesis [9]. The program was used to calculate measurements for Kambin’s triangle (i.e., an area defined by the superior articulating facet, exiting superior nerve root, and superior endplate of the inferior vertebral body) and transfacet corridors (i.e., the area between the superior articulating process and inferior articulating process), lending insight into the largest possible cannula size and the decision to proceed with a right-sided transfacet minimally invasive transforaminal lumbar interbody fusion. Similarly, CT imaging can be loaded onto robotic software such as Microsoft HoloLens to determine screw size, entry points, and trajectories for pedicle screw instrumentation [10]. Other popular AR devices approved for preoperative use and simulation have been shown to reduce errors in the training and simulation environments, but head-mounted displays are still of limited use in intraoperative settings [5,11].

AR guidance in real-time MISS is proven to be effective in improving accuracy, precision, efficiency, and safety. Integrating AR allows for greater navigation and spatial awareness, leading to fewer errors in procedures like pedicle screw placement. A systematic review noted that of 13 articles investigating screw placement accuracy, all but one had accuracy rates of at least 94% when graded with the Gertzbein and Robbins scale, a scale used to classify the accuracy of pedicle screw placement [8]. Notably, the first AR-assisted spinal surgery was a spinal fusion procedure conducted in 2020 with the XVision system [12]. Six pedicle screws were inserted with a clinical accuracy of 100%. Precision analysis revealed linear and angular deviations comparable to prior cadaver data. Furthermore, AR has been shown to reduce operation times, prompting superior recovery outcomes and fewer complications in patients [13]. Hu et al. (2020) compared a group of percutaneous vertebroplasty cases assisted with AR to traditional fluoroscopy cases, and they found a shorter procedural duration as well as more entry points [14]. Likewise, significantly shorter times have been observed in rod bending procedures using the HoloLens system [15].

AR in MISS can also reduce attention shift during operations since visualizations appear in the surgeon’s direct line of sight [13]. A study measuring attention shifts in different AR systems and traditional navigational tools revealed significantly fewer shifts with AR, regardless of the type of setup [16]. AR has been applied to a variety of MISS techniques, including in radiofrequency ablation for difficult-to-treat osteolytic lesions, puncture positioning procedures, screw placement in minimally invasive transforaminal lumbar interbody fusion, and more [17-19]. These new methods have mitigated high levels of occupational radiation exposure by replacing traditional fluoroscopy techniques [8].

Overall, current evidence suggests AR can achieve similar or greater results on performance measures compared to conventional systems. However, additional long-term data and large-scale clinical trials must be performed to validate the early success of AR in MISS.

Educational applications of AR in MISS

AR has demonstrated considerable potential, not only in enhancing surgical techniques and improving outcomes, particularly in MISS, but also as an effective teaching tool. Recent evidence underscores the value of AR platforms in significantly advancing surgical training and performance. For example, Gasco et al. (2014) found that using AR as a teaching aid in pedicle screw placement resulted in a 50% reduction in errors compared to traditional visual and verbal training methods [20]. In line with these findings, another study highlights the role of AR in improving the safety of pedicle screw placement despite lacking surgical experience. In this study, 10 candidates without surgical experience applied 36 pedicle screws using the C2-C3 posterior transpedicular fixation technique on 3D-printed vertebra models. The results revealed that 77.8% (14/18) of screws in the AR group were safely inserted, compared to 33.3% (6/18) in the freehand group (p = 0.018) [21]. Yu et al. (2019) found that a combination of virtual and AR aids in educating trainees for percutaneous transforaminal endoscopic discectomy [22]. This technology significantly reduces puncture and fluoroscopy times during training while also improving training effectiveness for young surgeons [22].

In addition to enhancing performance, safety, and efficacy in training, AR may affect the perceived difficulty of MISS. In Schmidt et al.’s (2024) study, 12 neurosurgical residents compared their experiences in simulation training performing minimally invasive transforaminal lumbar interbody fusion on a lumbar spine model with and without AR in the microscope [23]. The residents reported that their ability to maintain anatomical orientation and manage workload was better with AR, including significantly lower mental demand (p = 0.003) and perceived procedural difficulty (p = 0.019) [23].

AR additionally offers significant advantages for surgical training logistics, such as cost reduction by replacing expensive, nonreusable resources like cadavers and 3D-printed models in surgical training [8]. Thus, AR technology can expedite the learning process, allowing healthcare systems to train more surgeons in complex techniques efficiently. By shortening the learning curve, AR can enhance the proficiency of novice physicians, making training more accessible and cost-effective [5]. While still in its early stages in spinal surgery, AR shows great promise in improving surgical education and intraoperative performance.

Challenges and limitations

Barriers to the widespread adoption of AR in MISS include a combination of technological, logistical, and user-related challenges. Mechanical and visual discomfort associated with AR devices, such as head-mounted displays, may cause sensory overload due to mixing visual input with holographic data, resulting in fatigue during prolonged procedures [24]. Furthermore, adapting to AR-assisted workflows requires familiarity with the technology and integrating it seamlessly into traditional surgical techniques. This challenge is heightened for surgeons not accustomed to AR interfaces.

Financial barriers further hinder AR adoption, as some systems rely on intraoperative cone beam CT imaging, which is expensive and inaccessible in resource-limited settings [25]. Moreover, the lack of standardized protocols for assessing clinical outcomes in AR-guided procedures remains a limitation. For instance, there is no universally accepted protocol to grade pedicle screw placement accuracy or safety [24]. Additionally, the accuracy of AR project overlays in the surgical field needs to continue to be evaluated in a variety of challenging clinical scenarios, such as irregular or complex anatomy, as inaccurate projections may potentially hinder its implementation in clinical workflows.

Despite these hurdles, advancements in real-time visual recognition and processing have improved AR systems, enabling automated segmentation of anatomical structures, real-time holographic overlays of patient anatomy, and precise tracking of surgical tools within the operative field. These innovations reduce attention shifts, line-of-sight interruptions, and reliance on ionizing radiation, making AR a promising tool for enhancing surgical accuracy and efficiency [25,26].

Future directions

Ongoing studies are exploring the use of AR and other navigation technologies in MISS. The Surgical Navigation or Free Hand Technique in Spine Surgery trial is evaluating the accuracy and safety of pedicle screw placement using AR surgical navigation compared to traditional freehand surgical techniques in patients with spinal deformities (NCT05107310) [27]. Accurate screw placement is critical in these cases to prevent vascular, neural, or pulmonary complications while ensuring optimal fixation for deformity correction. By incorporating AR, the study aims to reduce the need for revision surgeries.

Another study, the NeuroSuitUp project, focuses on neurorehabilitation in patients with cervical spinal cord injuries using brain-computer interfaces, robotic systems, and AR to promote dormant neuroplasticity (NCT05465486) [28]. Although primarily a rehabilitation study, its use of AR and man-machine interfaces has implications for MISS by advancing precision and understanding of CNS plasticity.

Additionally, researchers at Strasbourg University are developing an AR-based lumbar puncture simulator, which uses haptic feedback to replicate the sensation of puncturing the ligamentum flavum (NCT05269238) [29]. This innovation enhances procedural training by allowing clinicians to practice in a realistic yet controlled environment. These studies demonstrate the potential for AR to improve outcomes and address challenges associated with minimally invasive spinal procedures.

## Conclusions

AR guidance in MISS holds promise with advancements in real-time processing and the adoption of technology. By addressing current challenges, AR has the potential to revolutionize surgical precision and efficiency. Ongoing research and innovation on navigation systems, neurorehabilitation, and training simulators underscore the versatility of AR in spinal procedures. As these technologies mature, they are likely to become indispensable tools in the field.
